# Punicic Acid a Conjugated Linolenic Acid Inhibits TNFα-Induced Neutrophil Hyperactivation and Protects from Experimental Colon Inflammation in Rats

**DOI:** 10.1371/journal.pone.0006458

**Published:** 2009-07-31

**Authors:** Tarek Boussetta, Houssam Raad, Philippe Lettéron, Marie-Anne Gougerot-Pocidalo, Jean-Claude Marie, Fathi Driss, Jamel El-Benna

**Affiliations:** 1 INSERM, U773, Université Paris, Faculté de Médecine, Paris, France; 2 Assistance Publique Hopitaux De Paris (AP-HP), Centre Hospitalo-Universitaire Xavier Bichat, CIB Phenogen, Paris, France; 3 Université de Versailles Saint Quentin en Yvelines, Versailles, France; BMSI-A*STAR, Singapore

## Abstract

**Background:**

Neutrophils play a major role in inflammation by releasing large amounts of ROS produced by NADPH-oxidase and myeloperoxidase (MPO). The proinflammatory cytokine TNFα primes ROS production through phosphorylation of the NADPH-oxidase subunit p47phox on Ser345. Conventional anti-inflammatory therapies remain partially successful and may have side effects. Therefore, regulation of neutrophil activation by natural dietary components represents an alternative therapeutic strategy in inflammatory diseases such as inflammatory bowel diseases. The aim of this study was to assess the effect of punicic acid, a conjugated linolenic fatty acid from pomegranate seed oil on TNFα-induced neutrophil hyperactivation *in vitro* and on colon inflammation *in vivo*.

**Methodology and Principal Findings:**

We analyzed the effect of punicic acid on TNFα-induced neutrophil upregulation of ROS production *in vitro* and on TNBS-induced rat colon inflammation. Results show that punicic acid inhibited TNFα-induced priming of ROS production *in vitro* while preserving formyl-methionyl-leucyl-phenylalanine (fMLP)-induced response. This effect was mediated by the inhibition of Ser345-p47phox phosphorylation and upstream kinase p38MAPK. Punicic acid also inhibited fMLP- and TNFα+fMLP-induced MPO extracellular release from neutrophils. *In vivo* experiments showed that punicic acid and pomegranate seed oil intake decreased neutrophil-activation and ROS/MPO-mediated tissue damage as measured by F2-isoprostane release and protected rats from TNBS-induced colon inflammation.

**Conclusions/Significance:**

These data show that punicic acid exerts a potent anti-inflammatory effect through inhibition of TNFα-induced priming of NADPH oxidase by targeting the p38MAPKinase/Ser345-p47phox-axis and MPO release. This natural dietary compound may provide a novel alternative therapeutic strategy in inflammatory diseases such as inflammatory bowel diseases.

## Introduction

Inflammatory disorders such as inflammatory bowel diseases (IBD), rheumatoid arthritis, atherosclerosis, metabolic syndrome and ischemia/reperfusion injury are recognized as a major health problem worldwide [1], [2]. One common characteristic of these diseases is excessive production of pro-inflammatory mediators such as TNFα, GM-CSF, IL-1, IL-6, IL-8, leukotriene B4 and PAF, the presence of highly activated inflammatory cells such as neutrophils, monocytes and macrophages and excessive production of reactive oxygen species (ROS) [2]–[6].

Polymorphonuclear neutrophils play a key role in host defenses against invading microorganisms and excessive neutrophil activation participates to tissue damage in inflammatory disorders [5]–[9]. In response to a variety of agents, they migrate to the inflammatory site where they release proteases, bactericidal peptides and large quantities of ROS, a process known as the respiratory burst [9]. A major source of ROS in inflammatory lesions comes from the reduction of oxygen to the superoxide anion (O_2_^−^) by neutrophil NADPH oxidase, a multicomponent enzyme system [10] and by myeloperoxydase (MPO) which produces hypochloric acid from hydrogen peroxide [7]. In resting cells NADPH oxidase is inactive and its components are distributed between the cytosol and membranes. When cells are activated, the cytosolic components (p47phox, p67phox, p40phox and Rac2) migrate to the membranes and associate with gp91phox/NOX2 and p22phox which form the flavocytochrome b558 to assemble the catalytically active oxidase [10]–[13]. P47phox phosphorylation on several serines plays a pivotal role in oxidase activation in intact cells [14]–[16]. Neutrophil superoxide production can be potentiated by prior exposure to “priming” agents such as the pro-inflammatory cytokines TNFα, GM-CSF and IL-8 [17], [18]. These cytokines inherently induce a very weak oxidative response by neutrophils, but they strongly enhance neutrophil release of ROS upon exposure to a secondary applied stimulus such as bacterial *N*-formyl-methionyl-leucyl-phenylalanine (fMLP) [17]–[23]. Priming of neutrophils is an important process in inflammatory diseases since it participates to tissue injury involved in inflammation [17]. In healthy subjects, the activation of NADPH oxidase of neutrophils is tightly regulated to avoid tissue damage. We have recently found that phosphorylation of p47phox on Ser345 represents a critical mechanism for TNFα-induced priming of ROS production by neutrophils as shown by site-directed mutagenesis of Ser345 and use of a competitive inhibitory peptide containing the Ser345 sequence [18]. This peptide inhibited TNFα-induced priming of ROS production while preserving the physiological response to bacterial derived *N*-formyl peptides. The phosphorylation of this site is controlled by p38MAPKinase in TNFα-primed neutrophils. We have shown that this regulation can be impaired in chronic inflammatory states whereby neutrophils isolated from synovial fluids of rheumatoid arthritis patients are primed and are prone to release high amounts of ROS [18]. In agreement, their p47phox cytosolic subunit of NADPH oxidase was found to be phosphorylated on Ser345 and the competitive peptide decreased ROS hyperproduction by neutrophils [18].

Inflammatory bowel diseases (IBD) are systemic inflammatory disorders most commonly targeting gastrointestinal tract [24]. Such disease states as ulcerative colitis, Crohn's disease and infectious enterocolitis, involve neutrophil migration across intestinal epithelia, neutrophil accumulation in the inflamed intestine [24], [25], overproduction of inflammatory cytokines, and release of massive amounts of ROS from neutrophils [26], [27]. Both excessive production of ROS and release of degradative enzymes by neutrophils have been implicated in IBD [26], [27]. Therefore decreasing p47phox phosphorylation on Ser345 and subsequent ROS hyperproduction by neutrophils without altering neutrophil normal response to bacterial products, would represent a novel and safe strategy to develop new therapeutic anti-inflammatory agents.

Since conventional existing therapies against IBD remain partially successful, and often result in significant side effects for patients, novel and safer methods, including dietary manipulations, have been used as an alternative therapeutic or prevention strategy in IBD and other inflammatory diseases which involve PMN activation and oxidative stress. For example, dietary n-3 polyunsaturated fatty acids (PUFA), eicosapentaenoic acid (EPA) and docosahexaenoic acid (DHA), naturally present in fish oils have been widely used in animal models and in human clinical trials [28]. They have been shown to attenuate clinical signs of inflammation and neutrophil infiltration into inflamed tissues in an experimental model of colitis [29]. In presence of aspirin, n-3 PUFA are converted to active substances named resolvins and protectins which play a pivotal role in the resolution of inflammation [29]. However, the effects of n-3 PUFA on neutrophil functions remain controversial, depending on the doses supplemented and the experimental model used [30]–[33]. Recently, fatty acids with conjugated double bonds like conjugated linoleic acid (CLA) and conjugated linolenic acid (ClnA) have attracted considerable attention because of their suggested health benefits on inflammation and some cancers [34]–[37]. These conjugated fatty acids have been reported to reduce the incidence of mammalian tumors in mice, to inhibit proliferation and invasion of cancer cells in culture and to promote apoptosis of cancer cells [38], [39]. In western countries, CLA and ClnA are present only as trace fatty acids in dietary fats. In other countries around the Mediterranean sea, Iran, Afghanistan, India, China, and Japan, plants such as pomegranate (*Punica granatum*, Punicaceae), bitter gourds (*Momocardia charantia*), and other cucurbitacae, which contain high amounts of punicic acid a ClnA isomer (9Z,11E,13Z) containing cis-9, trans-11, cis-13 double bonds in the C18 carbon chain, are regularly consumed [34], [35]. One might speculate that intake of these specific conjugated fatty acids could account for the lower incidence of inflammatory diseases in these populations [40], [41].

The aims of this study were to investigate the effects of punicic acid on TNFα-induced ROS over-production by human neutrophils and to evaluate the effect of punicic acid in a rat model of 2, 4, 6-trinitrobenzenesulfonic acid (TNBS)-induced colitis. We found that punicic acid exerted a strong inhibitory effect on TNFα-induced priming of ROS production by neutrophils *in vitro*, through the inhibition of p47phox phosphorylation on the priming site Ser345 and the upstream kinase p38MAPK. Furthermore, oral administration of pure punicic acid, or pomegranate seed oil rich in punicic acid to rats prevented TNBS-induced colitis and lowered ROS-induced tissue damage. Punicic acid may have beneficial anti-inflammatory effects by down regulating neutrophil hyper-activation.

## Results

### Punicic acid inhibits TNFα-induced ROS overproduction by human neutrophils while preserving the bacterial N-formyl peptides beneficial functions

**Figure 1:**
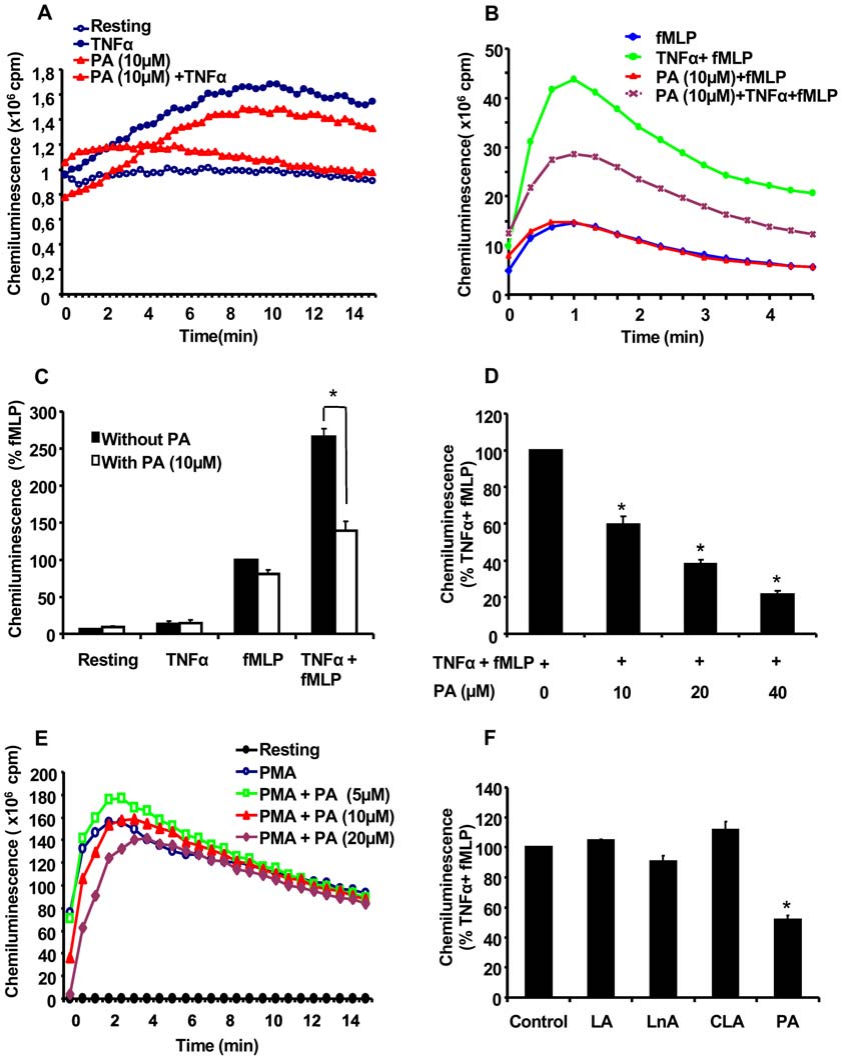
Punicic acid (PA) inhibits TNFα-induced ROS over-production by human neutrophils. (A) Human neutrophils (5×10^5^ cells in 0.5 ml Hanks buffer) were incubated in the absence or the presence of PA (10 µM) for 30 min; TNFα (10 ng/ml) was added and ROS production was measured by chemiluminescence technique in the presence of 10 µM luminol. (B) Human neutrophils (5×10^5^ cells in 0.5 ml Hanks buffer) were incubated in the absence or the presence of PA (10 µM) for 30 min; TNFα (10 ng/ml) was added for 20 min before stimulation with fMLP (10^−7^ M). ROS production was measured by chemiluminescence technique in the presence of 10 µM luminol. (C) Total chemiluminescence in each condition was quantified and presented as mean+/−SEM of 6 experiments (PA 10 µM), (* p<0.05 with versus without PA). (D) Dose effect of PA on TNFα-primed cells. Mean+/−SEM of 6 experiments of the dose effect of PA, * p<0.05 as compared to TNFα+fMLP controls (100%). (E) Dose effect of PA on PMA (100 ng/ml)-induced neutrophil ROS production. One representative of four experiments. (F) Human neutrophils (5×10^5^ cells in 0.5 ml Hanks buffer) were incubated in the absence or the presence of 10 µM of linoleic acid (LA), linolenic acid (LnA), conjugated linoleic acid (CLA) or PA for 30 min; TNFα (10 ng/ml) was added for 20 min before stimulation with fMLP (10^−7^ M). ROS production was measured by chemiluminescence technique in the presence of 10 µM luminol. Total chemiluminescence in each condition was quantified and presented as mean+/−SEM of 6 experiments, * p<0.05 as compared to TNFα+fMLP (control 100%).

We first tested the effect of punicic acid on TNFα-induced neutrophil priming of ROS production in response to fMLP. Neutrophils were incubated in the presence or absence of 10 µM of punicic acid for 30 min, treated in the presence or absence of TNFα, stimulated by fMLP and ROS production measured by luminol-amplified chemiluminescence. Our results show that in resting conditions and in the presence of TNFα alone, neutrophils produced a basal low level of ROS and punicic acid had no inhibitory effect on this process **(** **Figure 1A and 1C** **)**. The addition of fMLP resulted in the stimulation of ROS production, and as expected TNFα induced priming of fMLP-induced ROS production ; treatment of neutrophils with punicic acid prior to the addition of TNFα resulted in the inhibition of the priming effect of TNFα without altering the response induced by fMLP **(** **Figure 1B** **and** **Figure 1C** **)**. The effect of punicic acid was dose dependent starting from 10 µM up to 40 µM in final concentration **(Figure D)**. Punicic acid did not inhibit PMA-induced ROS production by neutrophils **(** **Figure 1E** **)**, suggesting that punicic acid did not inhibit NADPH oxidase activity nor scavenged ROS. At these concentrations punicic acid did not affect PMN viability as determined by the trypan blue exclusion method (data not shown). These results clearly show that punicic acid inhibited TNFα-induced ROS hyperproduction by neutrophils without affecting fMLP- nor PMA-induced responses.

We have also tested the effect of other fatty acids commonly found in edible oils such as linoleic acid (18 :2 ω 6), α linolenic acid (18:3 ω 3) and a conjugated linoleic acid (cis 9, 11 trans) compared to punicic acid. Results show (**Figure 1F**) that none of the tested fatty acids had an inhibitory effect on TNFα-induced priming of ROS production by neutrophils compared to punicic acid.

### Punicic acid inhibits TNFα-induced p47phox phosphorylation on Ser345 and p38MAPkinase phosphorylation

**Figure 2:**
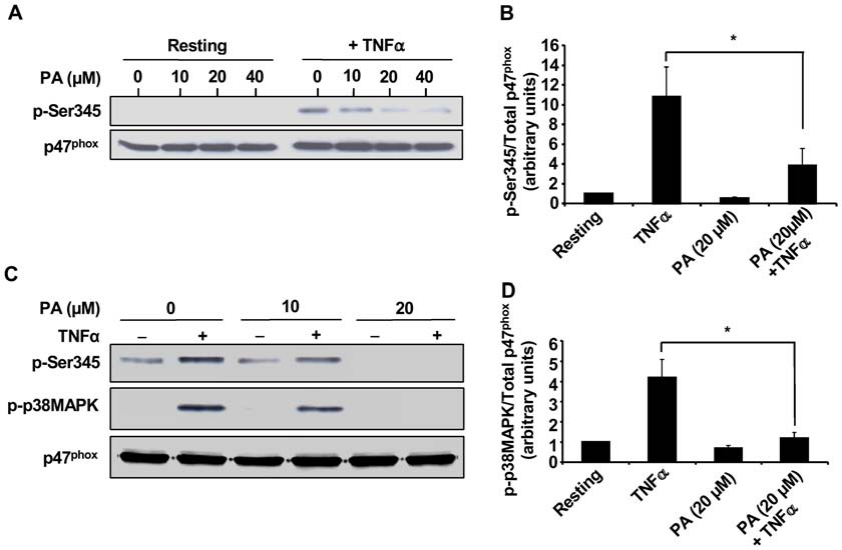
Punicic acid inhibits TNFα-induced p47phox phosphorylation on Ser345 and p38MAPKinase phosphorylation. (A) Neutrophils (1×10^7^ cells/ml) were pretreated with increasing concentrations of punicic acid (10 to 40 µM) for 30 minutes, then incubated with TNFα (10 ng/ml) or without (control) for 20 minutes. Cells were then lysed and proteins from 7×10^5^ cells were analyzed with SDS-PAGE and immunoblotting with anti-phospho-ser345 antibody (pSer345) or anti-p47phox antibody (p47phox). (B) Western blots from different experiments were scanned ; phosphorylated and total p47phox were quantified by densitometry , and the intensity of phosphorylated p47phox was corrected for the amount of p47phox. Results are expressed as mean+/−SEM (n = 3), * p<0.05 as compared to TNFα alone. (C) Neutrophils (1×10^7^ cells/ml) were pretreated with increasing concentrations (0, 10, 20 µM) of punicic acid for 30 minutes, then incubated with or without TNFα (10 ng/ml) for 20 minutes. Cells were then lysed and proteins from 7×10^5^ cells were analyzed with SDS-PAGE and immunoblotting with anti-phospho-ser345 antibody (pSer345), anti phospho p-38 MAPK (P-P38MAPK) and anti-p47phox antibody (p47phox). (D) Western blots from different samples treated or not with TNFα with or without 20 µM punicic acid were scanned, phosphorylated p38MAPK and total p47phox were quantified by densitometry and the intensity of phosphorylated p38-MAPK was corrected for the amount of p47phox. Data are representative of 3 independent experiments using cells from different healthy donors. Results are expressed as mean+/−SEM (n = 3). * p<0.05 as compared to TNFα alone.

Priming of neutrophil ROS production by the pro-inflammatory cytokine TNFα is mediated by the phosphorylation of p47phox on Ser345 [18]. We then analysed the effect of punicic acid on this process using a specific antibody directed against this phosphorylated site. Results show that punicic acid inhibited TNFα-induced p47phox phosphorylation on Ser345 in a dose-dependent manner (**Figure 2A**). Western blot analysis using an antibody directed against non phosphorylated p47phox showed that the same amount of proteins was present for all conditions tested. Phosphorylated and total p47phox from several experiments were quantified by densitometry and the amount of phosphorylated p47phox corrected for the amount of p47phox. These data clearly show that punicic acid strongly decreased TNFα-induced p47phox phosphorylation starting from 10 µM concentration as compared to TNFα alone (**Figure 2B**). The phosphorylation of p47phox on Ser345 is mediated by p38MAPK in TNFα-primed neutrophils (18). We therefore tested the effect of punicic acid on TNFα-induced p38MAPK phosphorylation. Results show that punicic acid inhibited p38MAPK phosphorylation paralleling the inhibition of p47phox phosphorylation on Ser345 with almost no detectable Phospho-Ser345 and phospho-P38MAPK at 20 µM punicic acid (**Figure 2C and 2D**). These results suggest that punicic acid inhibited TNFα-induced priming of ROS production by neutrophil NADPH oxidase by targeting the p38MAPKinase/Ser345-p47phox-axis.

### Punicic acid Inhibits TNFα- and fMLP -induced MPO release from neutrophils

**Figure 3:**
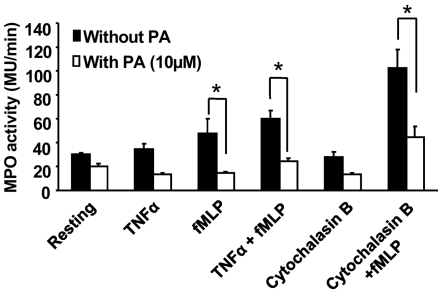
Effect of punicic acid on neutrophil degranulation. Human neutrophils from healthy donors were pretreated (open bars) or not (black bars) with punicic acid for 30 min, challenged with TNFα or cytochalasin B, then stimulated with fMLP for 3 min. Cells were centrifuged and MPO activity was determined in the supernatants using H_2_O_2_ and TMB as described in Materials and Methods. MPO activity was expressed in MU/min relative to a standard curve established with a known number of neutrophils. Data are representative of 4 different experiments. * P<0.05 vs untreated cells.

Superoxide anion (O_2_^−^) produced by NADPH oxidase is the source of other ROS generated by neutrophils. O_2_^−.^is transformed into H_2_O_2_ by spontaneous dismutation at acidic pH in the phagosome or in the extracellular medium. Myeloperoxidase (MPO) released from azurophilic granules catalyses the transformation of H_2_O_2_ in the presence of a halogen (Cl^−^,Br^−^, I^−^) into highly toxic molecules such as hypochloric acid (HOCl). Other reactions between OCl^−^ and H_2_O_2_ can lead to the formation of singlet oxygen (^1^O_2_). Most of the hypochloric acid thus generated is converted into toxic chloramines. Thus NADPH oxidase activation and MPO degranulation act in synergy during the inflammatory process. To test whether punicic acid could also affect neutrophil degranulation, human neutrophils were treated or not with punicic acid and MPO release was assessed by measuring its activity. As shown in **Figure 3**, TNFα alone did not induce MPO release in agreement with the literature (42), however it enhanced fMLP-induced degranulation. Cytochalasin B used as a positive control also enhanced fMLP-induced degranulation. Punicic acid inhibited fMLP and TNFα+fMLP- and cytochalasin B+fMLP-induced degranulation of azurophilic granules, resulting in less MPO release by neutrophils. Punicic acid had no direct effect on MPO enzymatic activity (data not shown). Taken together these results indicate that punicic acid inhibited TNFα-priming of both neutrophil NADPH oxidase activation and MPO release, and thus it may have an anti-inflammatory activity.

### Punicic acid inhibits TNFα-induced priming of ROS production by rat neutrophils and protects rats from TNBS-induced colon inflammation

Neutrophils and TNFα are two major elements known to be involved in inflammatory bowel diseases (IBD) and excessive production of ROS and release of degradative enzymes by neutrophils have been implicated in IBD [26], [27]. The results described above suggest that punicic acid could have anti-inflammatory effects, therefore we wanted to test the effect of punicic acid in an animal model of inflammatory bowel diseases. Studies have shown that among animal models of intestinal inflammation, TNBS induces macroscopic and histological features in rats or mice, similar to those occurring in human IBD and characterized by the presence of high number of activated neutrophils [43].

**Figure 4:**
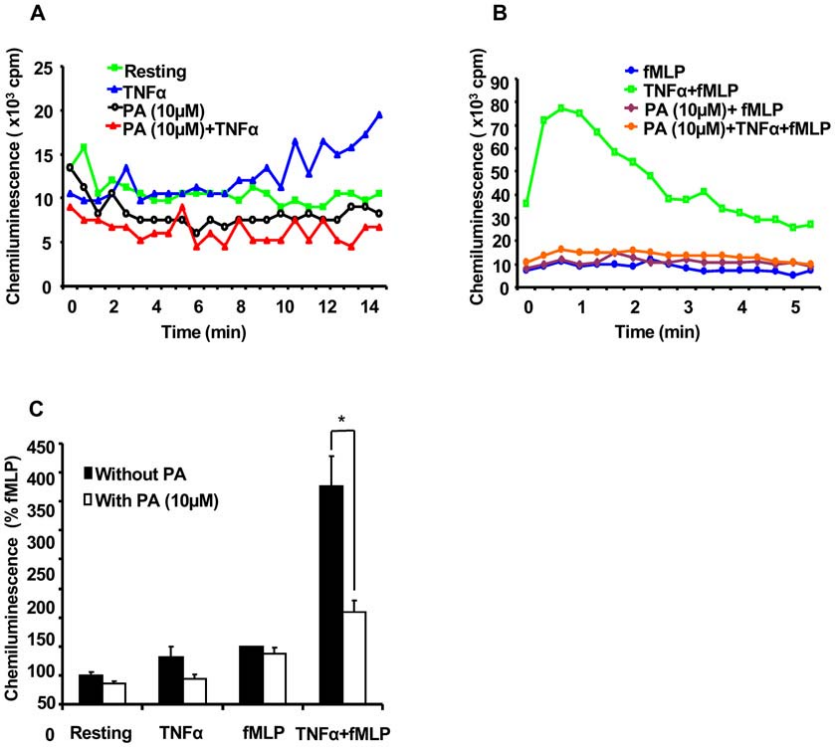
Punicic acid (PA) inhibits TNFα-induced ROS over-production by rat neutrophils. (A) Rat neutrophils (5×10^5^ cells in 0.5 ml Hanks buffer) were incubated in the absence or the presence of PA (10 µM) for 30 min; TNFα (10 ng/ml) was added and ROS production was measured by chemiluminescence technique in the presence of 10 µM luminol. (B) Rat neutrophils (5×10^5^ cells in 0.5 ml Hanks buffer) were incubated in the absence or the presence of PA (10 µM) for 30 min; rat TNFα (10 ng/ml) was added for 20 min before stimulation with fMLP (10^−7^ M). ROS production was measured by chemiluminescence technique in the presence of 10 µM luminol. (C) Total chemiluminescence in each condition was quantified and presented as mean+/−SEM of 3 experiments (PA 10 µM). * P<0.05 treated vs untreated cells.

In order to validate the model, we first tested the effect of punicic acid on TNFα-induced priming of ROS production by rat neutrophils. Rat neutrophils were incubated in the presence or absence of 10 µM of punicic acid for 30 min, treated in the presence or absence of rat TNFα then stimulated by fMLP. ROS production was then measured by luminol-amplified chemiluminescence. As expected, results show that TNFα alone and fMLP alone, induced low level of ROS production, and punicic acid had only a moderate inhibitory effect on this process **(** **Figure 4A, 4B and 4C** **)**. However, treatment of rat neutrophils by TNFα and their subsequent stimulation with fMLP resulted in enhanced ROS production. Treatment of neutrophils with punicic acid prior to the addition of TNFα resulted in the inhibition of ROS production by rat neutrophils (**Figure 4B and 4C**), suggesting that punicic acid inhibited the priming effect of TNFα. We secondly, showed that TNFα induced phosphorylation of p47phox on Ser345 in rat neutrophils and punicic acid inhibited this process (data not shown). These results suggest that, as observed in human neutrophils, punicic acid inhibited TNFα-induced priming of ROS production by targeting the p38MAPKinase/Ser345-p47phox-axis in rat neutrophils.

**Figure 5:**
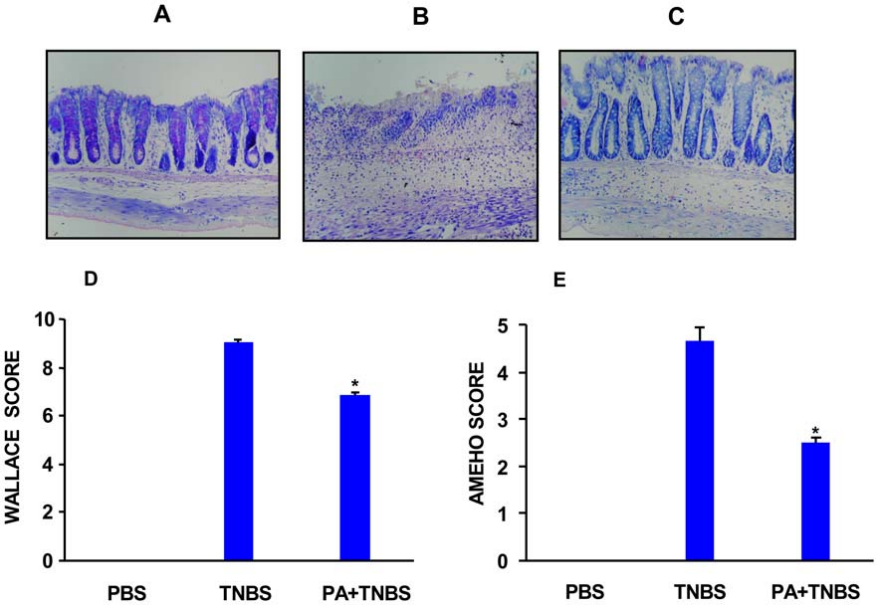
Effects of punicic acid on TNBS-induced colitis. Rats were gavaged with punicic acid (PA) dissolved in PBS (400 µg/0.5 ml) or PBS alone (0.5 ml) once a day for 10 days before the initiation of TNBS treatment. For the induction of colitis, rats were anesthetized, then they received an intrarectal administration of TNBS (250 µl, 150 mg/Kg) dissolved in 50% ethanol in 0.9% NaCl. Control rats received the vehicle ethanol or a saline solution using the same technique as described previously. Animals were sacrificed 2 days after TNBS administration. Macroscopic and histological analysis of colons were evaluated blindly by two investigators. Photomicrographs (magnification ×40) are representative of H&E stained slides of paraffin embedded colonic tissues recovered from (A) controls, (B) TNBS alone and (C) TNBS plus PA . Histologic and macroscopic lesions following TNBS treatment are represented by Wallace score (D) and Ameho score (E) as described in Materials and methods. The tissues are representative of 8 rats from each experimental group (* p<0.01 between TNBS and PA+TNBS).

We then used a rat model challenged with TNBS to examine whether dietary supplementation with punicic acid would prevent intestinal inflammation. In this model, TNBS was administered intra-rectally to induce an immunological response similar to the one found in human Crohn's disease [44]. Rats were daily gavaged with free punicic acid or PBS during 10 days before TNBS treatment. **Figure 5A** depicts a representative section of colon from untreated rats, while **Figure 5B** depicts a representative section of colon from rats 48 h after TNBS administration. As shown in these figures, the colon from TNBS-treated versus vehicle-treated rats displayed an extensive ulceration with cellular necrosis, oedema and inflammatory infiltrate. Colons from rats gavaged with punicic acid and treated with TNBS were characterized by a moderate inflammatory infiltrate with only punctuate mucosal erosion, indicating an important reduction of damage to the colon (**Figure 5C**). The representative histological features encountered for TNBS and punicic acid-TNBS treated rats were examined by two independent pathologists. As shown in **Figure 5D and 5E**, punicic acid induced a significant improvement in the macroscopic and histological scores evaluated according to the multi-parametric Wallace and Ameho criteria.

**Figure 6:**
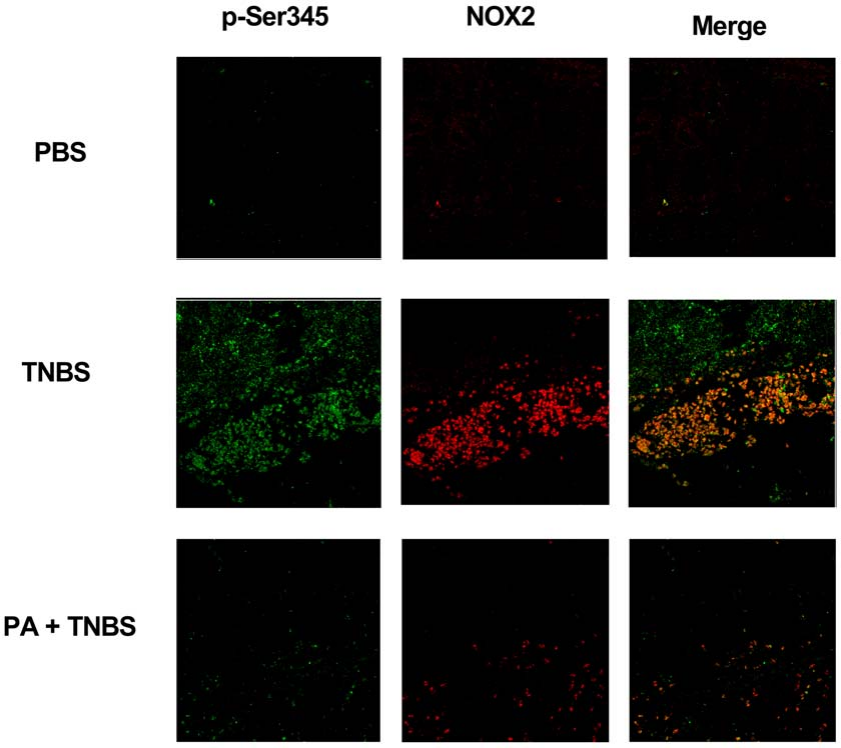
Punicic acid inhibits the phosphorylation of p47phox on Ser345 in vivo. Rats received punicic acid dissolved in PBS (400 µg/0.5 ml) or PBS alone (0.5 ml) once a day for 10 days before TNBS treatment. Rats were anesthetized, then they received an intrarectal administration of TNBS (250 µl, 150 mg/Kg) dissolved in 50% ethanol in 0.9% NaCl . Control rats received only 50% ethanol vehicle. Animals were sacrificed 2 days after TNBS administration. Colons were fixed in formalin and paraffin-embedded tissue sections (5 µm) and were analyzed by confocal microscopy as indicated in the Methods section. The tissues were incubated overnight at 4°C with rabbit anti-phospho-Ser345p47phox polyclonal antibody (1∶1000), and mouse anti-gp91phox monoclonal antibody (1∶1000) diluted in 1% BSA/PBS. Following this incubation, the tissues were washed four times in PBS and incubated with Alexa Fluor 488-(green) conjugated goat anti-rabbit antibody (1∶200) diluted in 1% BSA/PBS and Alexa Fluor 568 (red) conjugated goat anti mouse (1∶200) for 1 h at room temperature in the dark. Stained cells were examined with a 63/1.4 numerical aperture objective under a Zeiss LSM510 confocal microscope and the images were imported into an LSM image browser for analysis. Merge corresponds to colocalisation of NOX2 and P-Ser345 as described in materials and methods.

To identify if the protective effect of punicic acid was due to its inhibitory action on neutrophil excessive activation, we analyzed *in vivo* neutrophil priming in colon sections by confocal microscopy using the anti-phosphoSer345-antibody. NOX2 antibody was used to detect total phagocytic NADPH oxidase. Confocal analysis clearly showed (**Figure 6**) that control untreated rat colon (**Figure 6** **-PBS**) expressed low NOX2 and phospho-Ser345 (p-Ser345) fluorescence intensity in accordance with few neutrophil infiltration while TNBS treatment induced bright NOX2 and p-Ser345 fluorescence intensity in accordance with massive neutrophil infiltration and priming or activation as assessed by the colocalization of anti-gp91phox/NOX2 and the anti-phosphoSer345-antibodies, respectively (**Figure 6** **, p-Ser345, NOX2, Merge**). Punicic acid significantly reduced neutrophil infiltration and priming/activation in the colon of TNBS-treated rats, as shown by decreased NOX2 and p-Ser345 fluorescence intensity compared to TNBS-treated rats that did not receive punicic acid.

**Figure 7:**
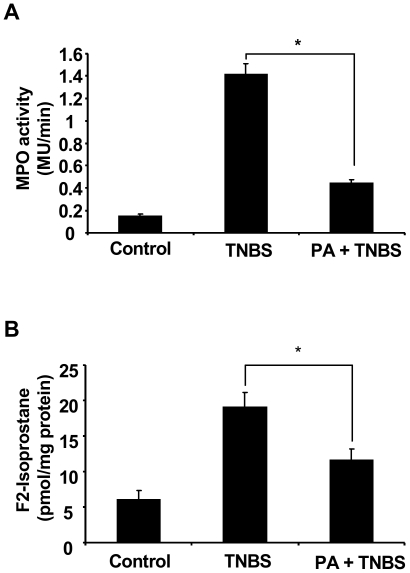
Punicic acid decreases TNBS induced MPO activity and F2- isoprostanes production in rat colons. Rats were treated as described in Figure 4 and 5. MPO activity (A) was defined as that degrading 1 µmol of hydrogen peroxide per g of tissue per minute. F2-isoprostane levels (B) was measured as described in Materials and methods. Data are expressed as mean+/−SEM of 6 rats in each group. (* p<0.05).

Neutrophil hyper-activation often results in excessive ROS production and degranulation which release high amounts of enzymes such as MPO resulting in lipid peroxidation and tissue injury. In this study, we measured MPO activity and F2-isoprostane the most reliable in vivo lipid peroxidation marker [45], in colons of control and punicic acid/TNBS challenged rats. **Figure 7A and 7B** show that TNBS induced a concomitant increase in MPO activity and F2-isoprostane production, as compared to control rats receiving only vehicle (PBS). Oral intake of punicic acid significantly decreased both MPO and F2-isoprostane levels. These data suggest that punicic acid has an anti-inflammatory effect through neutrophil inhibition of MPO and ROS release resulting in a decrease in lipid peroxidation and tissue injury.

### Pomegranate seed oil intake protects rats from TNBS-induced colon inflammation

**Figure 8:**
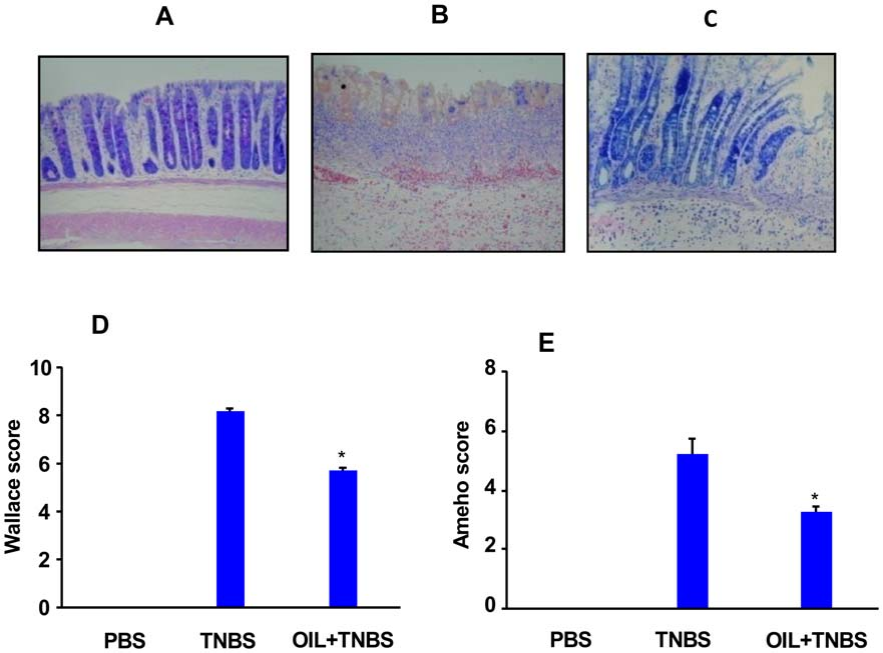
Effect of pomegranate seed oil on TNBS-induced colitis in rats. Photomicrographs (magnification ×40) were representative of H&E stained slides of paraffin embedded colonic tissues recovered from (A) controls, (B) TNBS alone and (C) TNBS plus pomegranate seed oil. Histologic and macroscopic lesions following TNBS treatment are represented by Wallace score (D) and Ameho score (E) score. The tissues are representative of 8 rats from each experimental group. Rats treated with oil show lower scores as compared to rats which received TNBS only. (* p<0.01).

Pomegranate seed oil is a natural source of punicic acid which comprise 80% of its total fatty acid content [46], we therefore evaluated if dietary supplementation of this oil has the same protective effect on TNBS-induced inflammation as pure punicic acid. It has been reported that pomegranate seed oil is efficient in vivo when it is used between 1% and 5% by weight of diet for a period of 3 to 32 weeks (39,46). We thus used a dose of 2% pomegranate seed oil by weight of diet for 10 days in our study. For this purpose, rats were daily gavaged with 0.5 ml of pomegranate seed oil for 10 days before TNBS treatment. **Figure 8A** depicts a representative section of colon from untreated rats, while **Figure 8B** depicts a representative section of colon from rats 48 h after TNBS administration. As shown in these figures, the colon from TNBS-treated versus vehicle-treated rats displayed an extensive ulceration with cellular necrosis, oedema and inflammatory infiltrate. Colons from rats gavaged with pomegranate seed oil and treated with TNBS were characterized by a moderate inflammatory infiltrate with only punctuate mucosal erosion, indicating an important reduction of damage to the colon (**Figure 8C**). The representative histological features encountered for TNBS and pomegranate seed oil-TNBS treated rats were examined by two independent pathologists. As shown in Figure 8D and 8E, pomegranate seed oil induced a significantly improvement in the macroscopic and histological scores evaluated according to the multi-parametric Wallace and Ameho criteria. Taken together, our results provide evidence that punicic acid/or pomegranate seed oil could have anti-inflammatory effect and may have a promising protective effect against inflammatory related diseases.

## Discussion

In the present study we show that punicic acid, a conjugated fatty acid, is a strong inhibitor of TNFα-induced priming of ROS production and MPO release by neutrophils. The inhibition of TNFα-induced priming of NADPH oxidase is the result of the inhibition of the phosphorylation of p38MAPKinase and p47phox on Ser345. The results also show that pure punicic acid and pomegranate seed oil protected rats from TNBS-induced colon inflammation. This protection could be the result of the inhibition of ROS/MPO-induced tissue damage as shown by the reduced level of lipid peroxidation product F2-isoprostane. We conclude that punicic acid could exert potent anti-inflammatory effects through: 1) the inhibition of TNFα-induced p38MAPK and p47phox-Ser345 phosphorylation in neutrophils, resulting in the inhibition of ROS excessive release and 2) the inhibition of MPO release, thus resulting in low tissue damage. This natural dietary compound may provide a novel alternative therapeutic strategy for inflammatory diseases which involve oxidative stress such as IBD, rheumatoid arthritis and atherosclerosis.

Proinflammatory cytokines play a key role in the pathophysiology of IBD [47] and anti-TNFα antibodies are therapeutically used in some severe forms of IBD [48]. TNFα is involved in priming of neutrophil ROS production which is a key process in inflammatory reaction (17). The presence of primed neutrophils was described in various inflammatory diseases such as rheumatoid arthritis, IBD, coronary heart disease and diabetes [18], [49], [50]. One of the molecular mechanism underlying this priming state of neutrophil-ROS production is the phosphorylation of p47phox on Ser345. A potential anti-inflammatory therapeutic intervention would be to use nutritional agents to decrease the phosphorylation of this site and subsequent ROS overproduction in inflammatory diseases without altering normal ROS production which is necessary for innate immunity and host defense. In the present study, we show that punicic acid, a naturally occurring fatty acid present in the diet of humans, was able to inhibit TNFα-induced priming state and reduce ROS production by human neutrophils without affecting ROS production induced by the bacterial chemotactic peptide fMLP or the PKC activator PMA. This inhibitory effect was mediated by the inhibition of p47phox phosphorylation on Ser345 and the phosphorylation of the upstream kinase p38MAPK. Therefore, punicic acid did not inhibit NADPH oxidase activity itself, but rather exerted an anti-priming effect by preventing excessive NADPH oxidase activation and subsequent ROS derived oxidative stress. This is the first natural compound known to reduce TNFα-induced phosphorylation of Ser345-p47phox and the priming process.

We also showed that punicic acid inhibited MPO degranulation by neutrophils. As the presence of MPO and ROS (especially H_2_O_2_) at the inflammatory site generates toxic molecules, the inhibitory effect of punicic acid on MPO degranulation further support the potential action of this compound as an anti-inflammatory agent. The mechanisms of this inhibition are not known and will be the subject of further investigation in our laboratory. However *in vitro* studies showed that punicic acid had no effect on fMLP-induced migration of neutrophils (data not shown), suggesting that it could inhibit neutrophil toxic activities without affecting its beneficial functions required for host defense.

Other fatty acids have different effects on neutrophil ROS production. Linoleic acid, which is the major fatty acid consumed in western countries, has been shown to act synergistically with TNFα to activate neutrophil ROS production [51]. Linoleic acid long chain derivative, arachidonic acid can activate directly neutrophils [52] and can be enzymatically converted into pro-inflammatory eicosanoids such as LTB4, and PGE2 [53]–[55], which are chemo-attractants and strong activators of neutrophils. In contrast, long chain n-3 PUFA, such as EPA and DHA, mainly provided by fish oil, are converted to less inflammatory eicosanoids and have been reported to counteract the harmfull effects of n-6 PUFA eicosanoids [28]. Hardy et al demonstrated that pre-treating neutrophils with arachidonic acid, EPA and DHA enhanced their response to either fMLP, or PMA, thereby producing more superoxide than when challenged with the stimulators only [56]. Li et al reported that EPA and DHA caused augmented responses in TNFα-primed neutrophils [57]. However, none of these *in vitro* and *in vivo* experiments have investigated the molecular mechanism underlying this augmented ROS production. Studies have suggested that conjugated linoleic acid induced activation of PPAR gamma and delta and protected from experimental inflammatory bowel disease [58] ; however the effect of punicic acid on this mechanism is not known.

To test if punicic acid could have anti-inflammatory effect *in vivo*, we tested its effect in TNBS-induced inflammation, a well known model of bowel inflammation [59]. There is ample experimental and clinical evidence to suggest that the inflamed colon undergoes substantial oxidative stress by neutrophil derived oxidants [27], which represent a significant mechanism for tissue damage during chronic intestinal inflammation. The results presented in this study show for the first time the presence of primed neutrophils in the inflamed colon as detected by the anti phosphoSer345-p47phox antibody. Consistent with the marked mucosal ROS induced injury, rats treated with TNBS had significantly elevated levels of the lipid peroxidation product F2-isoprostane compared with controls. Punicic acid or pomegranate seed oil intake protected from TNBS-induced inflammation probably by inhibiting neutrophil priming and ROS-induced reactions. Punicic acid also inhibited neutrophil recruitment in this model. Since TNFα is not chemotactic for neutrophils and punicic acid affected moderately IL-8 and fMLP-induced neutrophil migration, the effect of punicic acid on neutrophil recruitment *in vivo* could be indirect. One possible explanation is that since TNFα-induced ROS production is known to control chemokine production via oxidant-dependent factors [60]–[62], punicic acid by inhibiting TNFα-induced excessive ROS production could inhibit ROS-mediated inflammatory reactions.

Since punicic acid is a major fatty acid of pomegranate seed oil, representing 60 to 80% of total fatty acids [46], we decided to use a dietary pomegranate seed oil as a supplement to rats, instead of pure punicic acid. *In vivo* studies indicated that given to rats in the triglyceride form, punicic acid was well absorbed, metabolized and stored in rat tissues [39], [46], [63], [64]. Pomegranate seed oil provided protection against TNBS-induced ulcerations and tissue damage. This protective effect of pomegranate seed oil could be attributed to punicic acid the major fatty acid of this oil which is in good agreement with our results obtained with pure punicic acid used as free fatty acid. However, it cannot be excluded that unknown minor compounds found in pomegranate seed oil could have some effects *in vivo*. Since, pomegranate seed oil provided protection against TNBS-induced inflammation; therefore, dietary intake of this oil could be protective for inflammatory diseases.

In humans, high amounts of EPA and DHA have been used to prevent inflammatory complications of various diseases and to reduce circulating levels of inflammatory markers [28]. There are reports from meta analysis studies showing a limited benefit from diet supplementation or manipulation with fish oils in IBD and RA [65] This may be due to the above described priming effect of these n-3 PUFA, which may minimize their inhibitory effect on arachidonic acid derived eicosanoid production.

In conclusion, this study shows that punicic acid *in vitro* inhibited TNFα-induced priming of neutrophil ROS production by targeting the p38MAPKinase/p47phox/NADPH oxidase axis and inhibited MPO release in the extracellular media. Furthermore, punicic acid and punicic acid rich pomegranate seed oil have an *in vivo* anti-inflammatory effect by limiting neutrophil activation and lipid peroxidation consequences. Punicic acid and pomegranate seed oil would be highly protective against inflammation and could be used for the prevention and treatment of various inflammatory diseases, such as inflammatory bowel disease, rheumatoid arthritis and coronary heart disease.

## Materials and Methods

### Reagents and antibodies

Punic acid was purchased from Larodan (Malmö, Sweden). Pomegranate seed oil was purchased from All organic trading (GmbH, Kempten, Germany). TNFα was from R&D Systems. TNBS, fMLP, PMA, protease and phosphatase inhibitors were from Sigma Chemical Co. Injection-grade water and 0.9% NaCl were endotoxin-free in the limulus test (Charles River). Endotoxin-free buffers and salt solutions were from Invitrogen. Anti phospho-Ser345-p47phox and anti-p47phox were previously described [18]. Anti phospho-p38MAPKinase was from Cell Signaling.

### Ethics Statement

Neutrophils were isolated from human blood from healthy volunteers with a written informed consent. All experiments were approved by the INSERM (Institut National de la Santé et de Recherche Médicale) institutional review board and ethics committee. Data collection and analyses were performed anonymously.

All animal work was conducted according to relevant national and international guidelines in accordance with the recommendations of the Weatherall report. All animal experiments were approved by the Committee on Animal Experimentation of INSERM and performed in compliance with the care and use of laboratory animals.

### Human neutrophil preparation and measurement of ROS production

Human neutrophils were isolated in LPS-free conditions by means of one step purification on polymorphprep gradient dextran sedimentation as previously described [18] . ROS production was measured by luminol-amplified chemiluminescence method: cells (5×10^5^) were suspended in 0.5 ml HBSS containing 10 µM luminol preheated to 37°C in the thermostated chamber of the luminometer (Berthold-Biolumat LB937). After a baseline reading, cells were treated by punicic acid, TNFα then stimulated with 10^−7^ M fMLP and chemiluminescence was recorded.

### Detection of Ser345 and p38MAPKinase phosphorylation in neutrophils by a specific antibody

Neutrophils (1×10^7^ cells/ml) were incubated in the absence or presence of different concentrations of punicic acid for 30 min, then treated with TNFα (10 ng/ml) for 20 min. Cells were lysed and proteins were denatured in Laemmli's sample buffer [66]. The samples were subjected to SDS-polyacrylamide gel electrophoresis (PAGE) in 10% polyacrylamide gels, and Western-Blot using standard techniques. Immunoblotting were performed with primary rabbit polyclonal antibodies : anti-phospho-Ser345 antibody (1/10 000 dilution) or anti-p47phox antibody (1/5000 dilution) or anti-phospho p38MAPKinases (1/2000) then with HRPO-labeled-goat anti-rabbit antibody (1/10 000). The reaction was detected using ECL reagents.

### Measurement of MPO release from human neutrophils

MPO release was performed by measuring MPO activity using H_2_O_2_-dependent tetramethyl benzidine (TMB)-oxidation assay at 650 nm. Cells (5×10^6^) were suspended in 0.5 ml HBSS, preheated to 37°C in the water bath, cells were treated with punicic acid, TNFα, cytochalasin B then stimulated or not with 10^−7^ M fMLP. Neutrophils were rapidly centrifuged and MPO activity was measured in the supernatants. Total MPO activity was expressed in mU/min relative to a standard curve established with known quantities of triton X-100 treated neutrophils.

### Animal experiments

Different groups of 10 rats (male Wistar 200 g, Elevage Janvier, Le Genest-St-Isle, France) were used. Punicic acid in PBS (400 µg/0.5 ml) or PBS alone (0.5 ml) or pomegranate seed oil (0.5 ml) were orally administered once a day for 10 days before the initiation of TNBS treatment. For the induction of colitis, rats were anesthetized for 90–120 min, then they received an intrarectal administration of TNBS (250 µl, 150 mg/Kg) dissolved in a 1∶1 mixture of 0.9% NaCl with 100% ethanol. Control rats received a 1∶1 mixture of 0.9% NaCl with 100% ethanol or a saline solution using the same technique as described previously (61). Animals were sacrificed 2 days after TNBS administration. Macroscopic and histological evaluation of colitis were made blindly by two investigators. The colon of each rat was examined to evaluate macroscopic lesions according to the Wallace criteria. The Wallace score rates macroscopic lesions on a scale from 0 to 10 based on features reflecting inflammation such as hyperemia, thickening of the bowel and extent of ulceration. A colon specimen located 1 cm above the anus was used for histological evaluation according to the Ameho criteria. This grading is scaled from 0 to 6 and takes into account the presence of erosion, ulceration or necrosis and the depth and surface of lesions.

### Confocal microscopy

Formalin-fixed, paraffin-embedded colon sections (5 µm) on glass slides were deparaffinized in toluene and rehydrated through graded alcohol solutions. After washing in PBS, the tissues were permeabilized with 1% Triton-X/PBS/BSA for 10 min and blocked with goat serum (Dako A/S Denmark) for 1 hour. After washing, the tissues were incubated overnight at 4°C with rabbit anti-phospho-Ser345p47phox polyclonal antibody (1∶1000), and mouse anti-gp91phox monoclonal antibody (1∶1000) diluted in 1% BSA/PBS. Following this incubation, the tissues were washed four times in PBS and incubated with Alexa Fluor 488-(green) conjugated goat anti-rabbit antibody (1∶200) diluted in 1% BSA/PBS and Alexa Fluor 568 (red) conjugated goat anti mouse (1∶200) for 1 h at room temperature in the dark. After washing five times with PBS, cells were mounted using the Prolong Gold antifade kit following the manufacturer's instruction. Stained cells were examined with a 63/1.4 numerical aperture objective under a Zeiss LSM510 confocal microscope (Carl Zeiss, Heidelberg, Germany), and the images were imported into an LSM image browser (Carl Zeiss) for analysis.

### Measurement of isoprostanes and MPO activity

F2-isoprostanes are produced by a non cyclooxygenase free radical induced lipid peroxidation of membrane bound arachidonic acid. Mucosal F2-isoprostane concentrations were used as indexes of markers of lipid peroxidation induced by oxidative stress [45]. Total F2-isoprostane concentrations were determined in colonic mucosa with a commercially available enzyme immunoassay (EIA) kit (Cayman Chemical, An Abor,MI). Briefly, approximately 30 mg of freshly thawed tissue were added to 10 vol of chloroform-methanol (2/1, vol/vol) containing butylated hydroxytoluene and flushed with a gentle stream of nitrogen. The sample was vortexed and the lipid phase was extracted, evaporated to dryness under a stream of nitrogen and resuspended in methanol. F2-Isoprostanes were purified through an immuno-affinity column containing anti F2-isoprostane monoclonal antibody. F2-Isoprostanes were eluted off the column with pure ethanol. The ethanolic phase was evaporated to dryness under a stream of nitrogen, reconstituted in EIA buffer, and assayed according to the manufacturer's recommendations. Results are expressed as pg isoprostane/mg proteins. MPO activity on the homogenized colon tissue was measured using the same technique as described above.

### Statistical analysis

All results are expressed as means±standard error of the mean (SEM). Significant differences were identified with Student's *t* test; p values of <0.05 were considered significant.
